# Comprehensively prognostic and immunological analysis of VRK Serine/Threonine Kinase 1 in pan-cancer and identification in hepatocellular carcinoma

**DOI:** 10.18632/aging.205389

**Published:** 2023-12-28

**Authors:** Dongxing Chen, Wuhan Zhou, Jiafei Chen, Jingui Wang

**Affiliations:** 1 Department of Hepatobiliary Surgery, The First Hospital of Putian City, Putian, Fujian 351100, China; 2 Department of Clinical Medicine, Fujian Medical University, Fuzhou, Fujian 350108, China

**Keywords:** VRK1, prognosis, immunotherapy, pan-cancer, hepatocellular carcinoma

## Abstract

Background: VRK1 is a member of the vaccinia-related kinase (VRK) family of serine/threonine protein kinases, which is related to the occurrence and development of malignant tumors. The expression pattern, predictive value, and biological function of VRK1 in various cancers remain largely elusive and warrant further investigation.

Methods: Public databases, such as TCGA, GTEx, and UCEC, were utilized to comprehensively analyze the expression of VRK1 across multiple cancer types. Prognostic significance was assessed through Univariate Cox regression and Kaplan-Meier analyses. Additionally, Spearman's correlation analysis was employed to explore the potential associations between VRK1 expression and various factors, including tumor microenvironment scores, immune cell infiltration, and immune-related genes. Moreover, to validate the findings, differential expression of VRK1 in HCC tissues and cell lines was further confirmed using qPCR, Western blot, and immunohistochemistry techniques.

Results: The upregulation of VRK1 was observed in most cancer types, and was associated with worse prognosis in ACC, KICH, KIRP, LGG, LIHC, LUAD, MESO, and PCPG. In various cancers, VRK1 expression exhibited positive correlations with immune infiltrating cells, immune checkpoint-related genes, TMB, and MSI. Furthermore, the promoter methylation status of VRK1 varied across different tumor types, and this variation was associated with patient prognosis in certain cancers. In our experimental analyses, we observed significantly elevated expression of VRK1 in both HCC tissues and HCC cells. Functionally, we found that the downregulation of VRK1 had a profound impact on HCC cells, leading to a significant decrease in their proliferation, migration, and invasion capabilities.

Conclusion: The expression of VRK1 exerts a notable influence on the prognosis of several tumors and exhibits a strong correlation with tumor immune infiltration. Moreover, in the context of HCC, VRK1 may act as an oncogene, actively promoting tumor progression.

## INTRODUCTION

Cancer represents a significant global health challenge, characterized by numerous genetic disorders, and has emerged as one of the primary causes of mortality worldwide [1]. Public databases, such as Cancer Cell Line Encyclopedia and The Cancer Genome Atlas, have been systematically analyzed and summarized to gain a better understanding of the pathogenesis of human malignancies [2]. The pan-cancer analysis is well established with the continued accumulation and development of multi-omics data across cancer types [3, 4]. More notably, unlike studies of single tumors, pan-cancer analysis reveals similarities and heterogeneity between different tumors. It provides a breadth of research and an integrated approach to cancer biology [5].

VRK1 is a member of the vaccinia-related kinase (VRK) family of serine/threonine protein kinases [6] and a nucleosome kinase or chromatin kinase [7]. In mechanism, VRK1 can directly bind to chromatin protein and participate in the cell cycle and apoptosis [8]. In addition, some studies have found that VRK1 can regulate various transcription factors to participate in the occurrence and development of tumors [9, 10]. Furthermore, HNRNP A1 could promote lung cancer cell proliferation by modulating VRK1 translation [11]. In Glioblastoma, knocking down VRK1 leads to decreased BAF activity, which in turn causes nuclear lobulation, blebbing, and micronucleation, resulting in G2-M arrest and DNA damage occurring as a result of these cellular alterations [12]. In breast cancer, overexpression of VRK1 can promote malignant progression of breast cancer cells [13]. In non-small cell lung cancer, Ginsenoside Rg3 regulates DNA damage by activating VRK1/P53BP1 pathway [14]. Downregulation of VRK1 suppressed the proliferative and migratory activity of esophageal cancer cells [15]. It is noteworthy that VRK1 is poised to exhibit diverse roles contingent upon the specific cellular type and its physiological or pathological context. The functionality of VRK1 is dual, as it can act as either an oncogenic driver, a tumor suppressor, or a gene predisposing to cancer [6, 16, 17]. Given the extensive involvement of VRK1 in a number of important biological processes such as cell cycle regulation, DNA repair, apoptosis and transcriptional regulation, the study of VRK1 in disease development and pan-cancer has attracted extensive attention in recent years. However, there is no research on VRK1 in pan-caner analysis. Therefore, this study aims to evaluate the role of VRK1 on various cancers and to analyze the relationship between VRK1 and the prognosis of tumor patients. In addition, our study aimed to delve into the specific biological functions of VRK1 in cancer through *in vitro* experiments. By gaining a deeper understanding of the functions and signalling pathways of VRK1, we expect to provide new targets and strategies for the treatment of diseases related to the cell cycle and invasive migration.

This analysis involved the integration of multiple databases to obtain a holistic understanding of VRK1’s expression patterns in different cancer types. Following the comprehensive pan-cancer analysis, we present essential insights into VRK1 across various cancer cohorts. We investigate the correlation between VRK1 expression and multiple factors, including prognosis, enriched gene sets, immune cell infiltration, and expression of immune regulators on a pan-cancer level. Reducing the expression of VRK1 significantly inhibited the occurrence and development of tumors in LIHC. Drawing from these data, we propose VRK1 as a novel and promising biomarker for predicting patient prognosis and gauging the potential effects of immunotherapy across various cancer types.

## METHODS

### Data collection

In our analysis, we examined the VRK1 mRNA expression levels in a wide range of tumor and normal tissues using datasets from The Cancer Genome Atlas (TCGA) and Genotype-Tissue Expression (GTEx), which were downloaded from the UCSC Xena database (https://xenabrowser.net/datapages/). The VRK1 protein expression was explored from The UALCAN portal (http://ualcan.path.uab.edu/index.html). For the MMR gene mutation and DNA methylation analysis, we utilized the Sangerbox online platform. To assess the correlation between VRK1 expression and the five MMR genes or four methyltransferases, we employed the Spearman’s correlation method. This approach allowed us to examine potential associations between VRK1 expression and the genetic and epigenetic factors related to MMR genes and DNA methylation. The abbreviations of the 33 studied cancers are shown in Supplementary Table 1.

### Analysis of genomic alterations

We explored the mutation landscape of VRK1 and gene mutation co-occurrence patterns between VRK1 signatures and other proteins from TCGA Pan-Cancer Atlas Studies in the different cancer types through the web tool cBioPortal (http://cbioportal.org) [18]. In the study, we visualized the mutated site information of VRK1 using a schematic diagram or a Three-dimensional structure representation, which was made available through the "Mutations" module. Furthermore, we conducted predictive analyses using the "Comparison" module to gain further insights into the functional implications of these mutations. This comprehensive approach allowed us to explore the potential impact of VRK1 mutations on its structure and function in the context of cancer.

### Analysis of epigenetic methylation

We employed the TCGA methylation module within the UALCAN interactive web resource. This analysis allowed us to compare the methylation patterns of VRK1 in tumor samples and their respective matched normal tissues, providing valuable insights into the epigenetic alterations associated with VRK1 in different cancer types [19, 20]. In addition, we analyzed the effects of methylation on dysfunctional T-cell phenotypes and prognoses using the Tumor Immune Dysfunction and Exclusion (TIDE)server.

### Tumor immune infiltration analysis

To investigate the relationship between VRK1 expression and immune infiltrates across all TCGA tumors, we utilized the "Immune-Gene" module available on the TIMER2 web server. This analysis allowed us to explore potential associations between VRK1 expression levels and immune cell infiltration in the context of various cancer types from the TCGA dataset. The correlations between VRK1 mRNA expression and 21 immune cell subsets were analyzed. A heatmap was generated to visualize the results of the purity-adjusted Spearman’s rank correlation test, providing *P*-values and partial correlation values. This analysis allowed us to explore the associations between VRK1 expression and other variables, taking into account tumor purity and provided valuable insights into the relationships among the studied parameters.

### VRK1-related gene analysis

To analyze the protein-protein interaction network of VRK1 according to the BioGRID (https://thebiogrid.org/), which is BioGRID is a biomedical interaction repository with data compiled through comprehensive curation efforts [21]. We applied the “Search BioGRID” module to perform a gene interaction analysis of VRK1. We used the “Network” module of ioGRID to display the network data of the interacted genes.

### Gene set enrichment analysis

Gene set enrichment analysis was employed to investigate the potential biological processes and mechanisms associated with VRK1. The “gmt” file of the hallmark gene set containing 50 hallmark gene sets was downloaded from the website of the Molecular Signatures Database. GSEA was conducted using the R package “clusterProfiler” [22], and the results were summarized in the bubble plot depicted by the R package “ggplot2”. Pathway analysis was then performed using Reactome Database (https://reactome.org/) [23].

### Immunotherapy prediction analysis

We utilized Spearman correlation analysis to assess the statistical associations between VRK1 and established immunotherapy biomarkers across different cancer types [24, 25]. The relationship between ICGs, TMB, and MSI was analyzed using the Sangerbox tool (http://www.sangerbox.com/tool). Additionally, we explored the correlation between VRK1 expression and the estimated proportions of immune and stromal cells through the Sangerbox online platform, which provided three types of scores: ImmuneScore, StromalScore, and EstimateScore. This comprehensive analysis allowed us to assess the potential associations between VRK1 expression and immune and stromal components in the tumor microenvironment.

### Survival prognosis analysis of VRK1

In this study, we utilized the survival analysis module in GEPIA2 to create a forest plot depicting VRK1’s overall survival (OS) and disease-specific survival (DSS) across all TCGA tumors. The expression levels were categorized into high and low expression cohorts using a cutoff value of 50%. Additionally, VRK1 expression data were further employed in a Kaplan-Meier curve analysis using the Sangerbox online platform.

### Cell lines, reagents, and plasmids

HCCLM3 and SK-HEP1 cell lines were procured from the Chinese Academy of Sciences Cell Bank in Shanghai, China. These cells were routinely cultured at 37°C in a humidified atmosphere with 5% CO_2_, using Dulbecco’s Modified Eagle Medium (DMEM) supplemented with 10% fetal bovine serum (Gibco, USA). To downregulate VRK1 expression, a plasmid encoding shRNA against VRK1 was designed and obtained from Genepharma Company, also located in Shanghai, China. The transfection of cells with either the shRNA or vector plasmids was carried out using Lipofectamine 3000, following the manufacturer’s instructions provided by Invitrogen.

### RNA extraction, cDNA synthesis, and real-time quantitative PCR

Total RNA was isolated according to the Trizol Reagent protocol. For the reverse transcription of RNA, the PrimeScript RT Reagent Kit from Invitrogen was employed. Subsequently, quantitative real-time PCR (qRT-PCR) was performed using the SYBR Green PCR Kit from Takara, located in Dalian, China. During qRT-PCR, aliquots of cDNA were amplified to quantify the gene expression levels of interest. Primer sequences were as follows: VRK1 (forward: 5′-GAGGCCATACAGACCCGTTC-3′; reverse: 5′-TCCACCTCGCAAGACTCACA-3′). GAPDH (forward: 5′-ACCCAGAAGACTGTGGATGG-3′; reverse: 5′-TTCTAGACGGCAGGTCAGGT-3′) was used as an internal control, and the expression level was calculated by using the (2^−ΔΔCT^) ratio.

### Cell proliferation, colony formation, and transwell assays

Several cell cultures were seeded in 96-well plates at 4.0 × 10^3^ cells/well concentrations. Cell viability was evaluated using the CCK-8 reagent at specified intervals, following the manufacturer’s protocol (KeyGEN). To explore cell proliferation, the Cell-Light EdU DNA Cell Proliferation Kit (RiboBio, Guangzhou, China) was used to calculate the ratio of EdU-positive cells to the total number of cells. Cells were transfected with the VRK1 shRNA plasmid for the colony formation assay and seeded in 6-well plates at 500 cells/well concentrations. After a 14-day incubation period, the plates were fixed, and a 0.5% crystal violet solution was used for staining. Transwell systems from BD Biosciences were utilized for conducting migration and invasion assays. Briefly, 6 × 10^4^ cells suspended in DMEM were seeded into the upper chambers without coating (migration) or with Matrigel (invasion). The lower chambers were filled with 15% FBS medium. The rest of the steps were carried out according to conventional experimental methods.

### Western blotting

In 10% SDS-PAGE gels, equal amounts of protein were loaded into each well. After separation, the proteins were transferred onto membranes, and the bands on the membranes were blocked using 5% FBS. Subsequently, the membranes were incubated with the respective primary antibodies overnight at 4°C. Subsequently, the bands were incubated with the secondary antibody and visualized using an ECL kit. For western blot assays, rabbit polyclonal anti-VRK1 and rabbit polyclonal anti-GAPDH antibodies were purchased and diluted to 1:1,000 and 1:2,000, respectively.

### Statistical analysis

In this study, the statistical association between VRK1 protein expression levels in clinical liver cancer samples and normal tissues was evaluated using a paired *t*-test. The predictive value of VRK1 expression in each cancer was assessed through the Kaplan-Meier method and univariate Cox regression analysis. To analyze differences between various groups, the student’s *t*-test or one-way ANOVA was applied. Additionally, Spearman’s correlation tests were utilized to determine the significance of correlations between VRK1 expression and immune cell infiltration, immune regulator expression, TMB, and MSI. The threshold for statistical significance for all two-sided statistical tests was set at *p* < 0.05. GraphPad Prism 8.0 was employed to analyze the experimental data, and statistical analyses were conducted using R 3.6.2. The results are presented as mean ± SD.

### Availability of data and materials

The data and materials of this article are included in the article/supplemental material. Any further questions should be forwarded to the corresponding author.

## RESULTS

### Basic Information on VRK1

**Figure 1:**
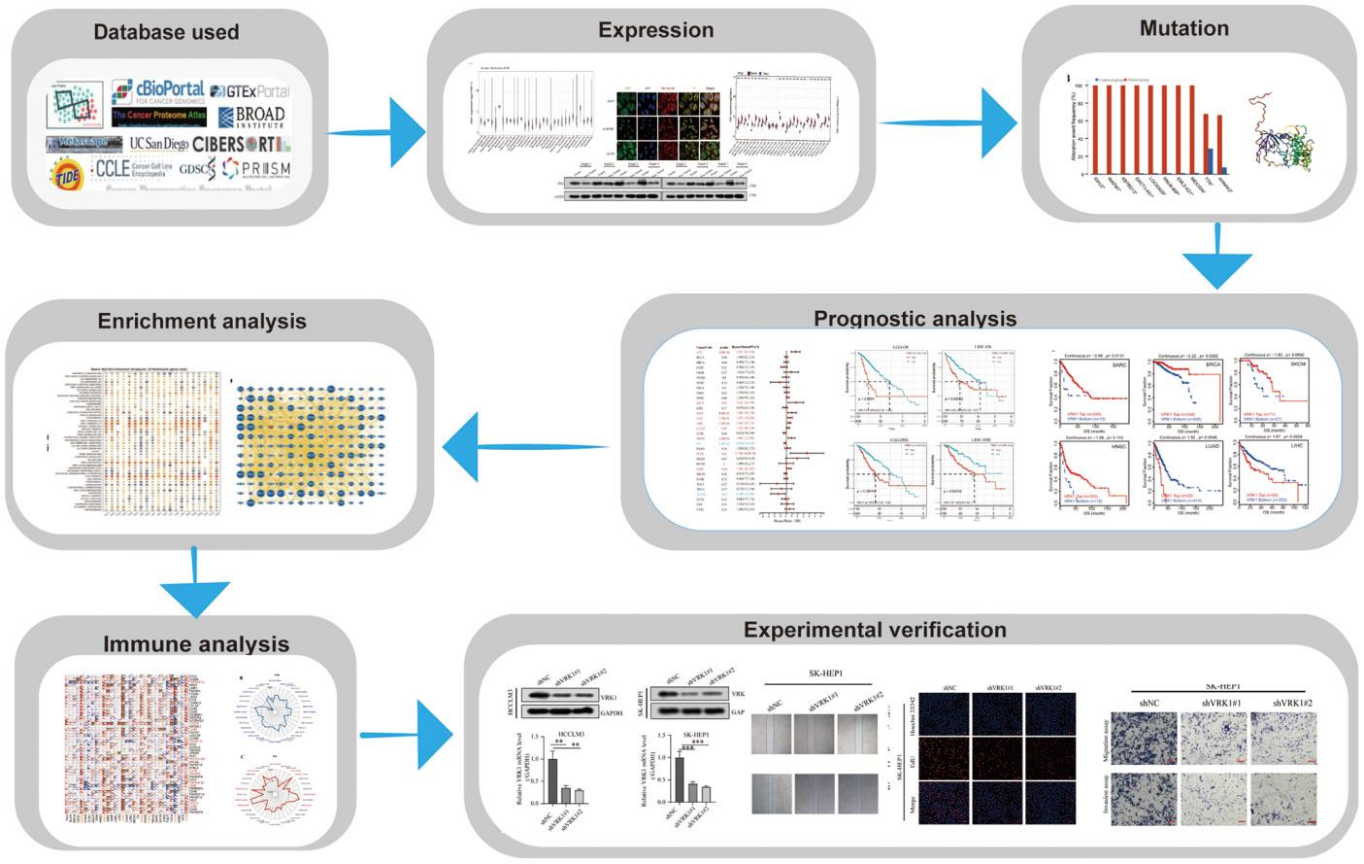
Flow chart of this article.

**Figure 2:**
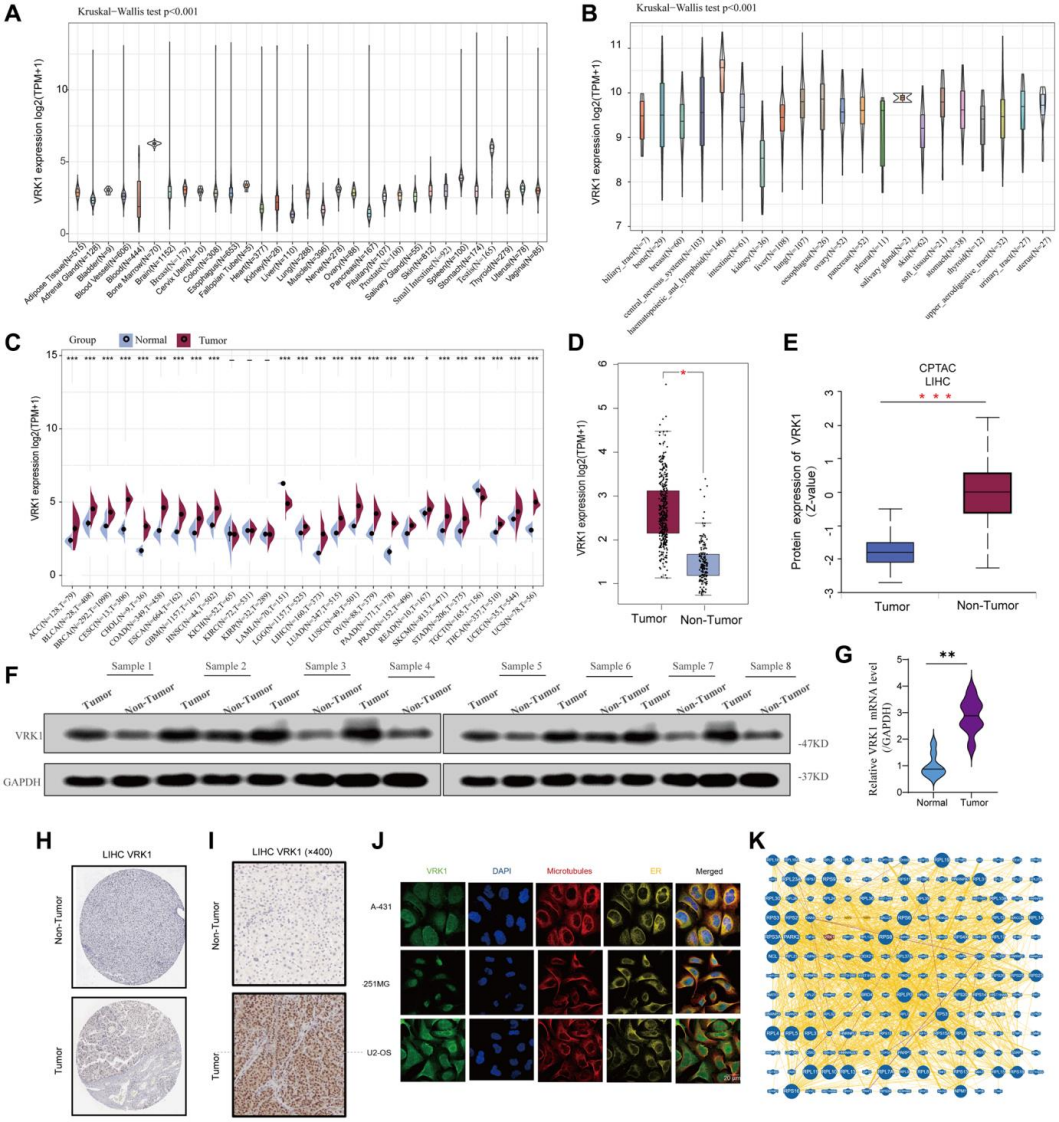
**Basic information of VRK1 expression.** (**A**) The different VRK1 expressions of the 31 types of cancers. (**B**) The different VRK1 expression in the cancer cell lines. (**C**) Comparison of VRK1 mRNA levels between cancerous and adjacent normal tissue from TCGA and GTEx datasets. (**D**) The mRNA level of VRK1 between LIHC and normal tissues. (**E**) The protein expression level of VRK1 between LIHC and normal tissues. (**F**, **G**) Western blotting and qRT-PCR assays explored the protein and mRNA expression level of VRK1 in the LIHC tissues and paired normal tissues. GAPDH was used as a loading control. (**H**, **I**) The expression level of VRK1 was detected using public databases and collected liver cancer tissue specimens by using IHC assay. (**J**) The immunofluorescence images of VRK1 protein, nucleus, endoplasmic reticulum (ER), microtubules, and the incorporative images in A-431, U-251MG, and U2-OS cell lines. (**K**) Protein–protein interactions of diagram of VRK1 protein (^*^P < 0.05, ^**^P < 0.01, and ^***^P < 0.001).

Figure 1 illustrates the flow chart of our study design. The results showed that VRK1 was heterogeneous and expressed at different levels in tissues and tumor cells (Figure 2A, 2B). Next, we evaluated the differential expression pattern of VRK1 in cancers and adjacent carcinoma tissues. As shown in Figure 2C, as depicted in Figure 2C, VRK1 exhibited high expression levels in a wide range of cancer types, including ACC, BLCA, BRCA, CESC, CHOL, COAD, ESCA, GBM, HNSC, LGG, LIHC, LUAD, LUSC, OV, PAAD, PRAD, READ, SKCM, STAD, THCA, UCEC, and UCS. In contrast, low VRK1 expression was observed in LAML and TGCT (Figure 2C). Compared with normal tissues, the mRNA and protein (Figure 2D, 2E) levels of VRK1 in LIHC were significantly upregulated. Overall, VRK1 mRNA levels were abnormal in various cancer types. To validate the findings of the informatics data analysis, we employed western blotting and qRT-PCR assays. These experimental approaches allowed us to confirm whether VRK1 protein levels were indeed aberrantly expressed in cancer samples. By comparing the experimental results with the informatics analysis, we sought to strengthen the evidence supporting the dysregulation of VRK1 in cancer. As expected, VRK1 expression was significantly upregulated in LIHC tissues than corresponding adjacent normal tissues (Figure 2F, 2G), consistent with IHC staining results (Figure 2H). And we also verified in the collected tissue specimens that VRK1 is indeed highly expressed in LIHC (Figure 2I). We also observed the correlation between VRK1 expression and the pathological stages of cancers, including ACC, BRCA, CESC, KICH, LIHC, SKCM, LUSC, and STAD (Supplementary Figure 1A). Next, VRK1 protein distribution at the subcellular level was determined using immunofluorescence images of cell lines. The results showed that VRK1 proteins were located at the ER and nucleus in the A-431, U-251-MG, and U2-OS cells (Figure 2J). Finally, a PPI network of VRK1 was constructed by the BioGRID database to identify the potential biological interactions (Figure 2K). We used the Reactome Pathway Database to identify signaling pathways associated with VRK1 and the co-expressed genes, including the immune system, cell cycle, signal transduction, and metabolism (Supplementary Figure 1B). In summary, VRK1 was highly expressed in most tumors.

### Mutation landscape of VRK1 in pan-cancer

**Figure 3:**
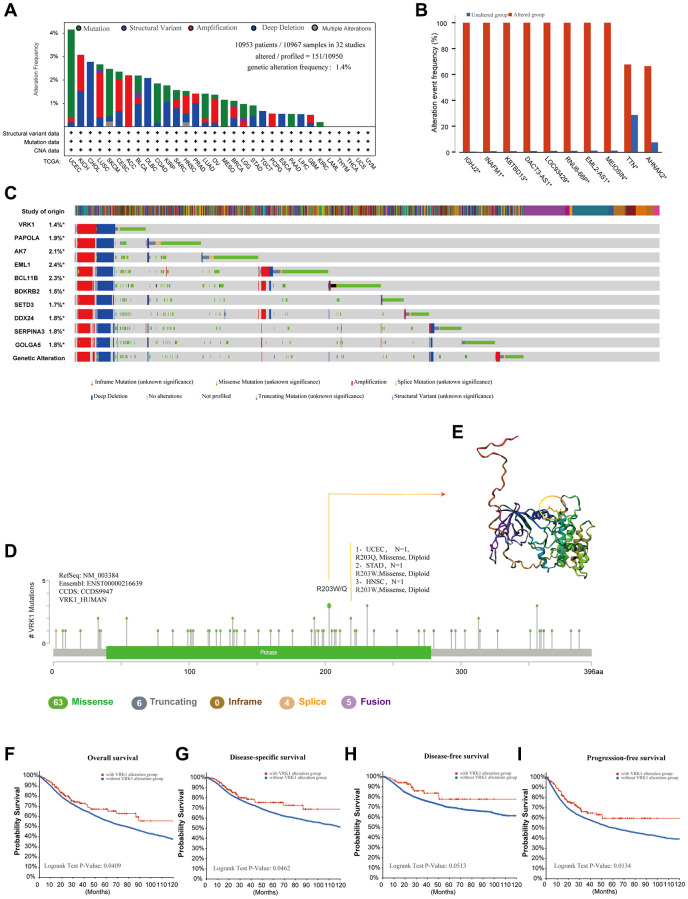
**Mutation information of VRK1 in pan-cancer.** (**A**) Histogram showing VRK1 alteration frequency and mutation type in various cancers. (**B**) Bar plot showing the frequencies of ten genes (IGHJ2, INAFM1, KBTBD13, DACT3-AS1, LOC93429, RNU6-66P, EML2-AS1, MEIOSIN, TTN, and AHNAK2) alteration cooccurrence with VRK1 alteration. (**C**) Waterfall plot showing the cooccurrence pattern of VRK1 alteration with genetic alterations of PAPOLA, AK7, EML1, BCL11B, BDKRB2, SETD3, DDX24, SERPINA3 and GOLGA5. (**D**) Mutation sites are displayed in the VRK1 structural domain. (**E**) The highest alteration frequency (R203W/Q) was displayed in the 3D structure of VRK1 (labeled in yellow). (**F**–**I**) The possibility of a link between VRK1 mutation status and overall survival (**F**), disease-specific survival (**G**), disease-free survival (**H**), or progression-free survival (**I**).

Next, we explored the mutation landscape of VRK1 in various cancers. As represented in Figure 3A, “deep deletion” was the primary alteration type in most cancers, followed by “mutation” and “amplification.” The highest VRK1 alteration frequency (>4%) appeared in the patients with UCEC with “Mutation” as the primary alteration type. Notably, some cancer types were observed only to have one kind of VRK1 genetic alteration. For example, CHOL, DLBC, TGCT, ESCA, and LIHC only exhibited deep deletion, while amplification was the only alteration type in ACC and PCPG. Furthermore, we observed that genetic alterations of VRK1 co-occurred with the frequency and pattern of these same genes, suggesting a functional partnership between VRK1 and these genes in the oncogenic processes across various cancer types (Figure 3B, 3C). These findings imply that VRK1’s role in cancer development might be influenced by its interactions with these specific genes, thus highlighting potential key players in the oncogenic pathways associated with VRK1. In Figure 3D, we provided a comprehensive overview of the specific sites and corresponding case numbers of VRK1 alterations. The findings showed that missense mutation was the primary type, R203Q was detected in the UCEC, inducing a missense mutation, and the mutations of R203W were found in the STAD and HNSC, resulting in a missense mutation. Also, we presented the 3D structure of R203W/Q in VRK1 (Figure 3E). Importantly, we analyzed to explore the potential association between genetic alterations of VRK1 and clinical survival prognosis. VRK1 alteration was associated with improved overall survival (OS), disease-specific survival (DSS), and progression-free survival (PFS) compared to patients without VRK1 alteration. However, there was no significant difference in disease-free survival (DFS) (Figure 3F–3I).

### Associations between VRK1 and immune regulators, TMB, and MSI

**Figure 4:**
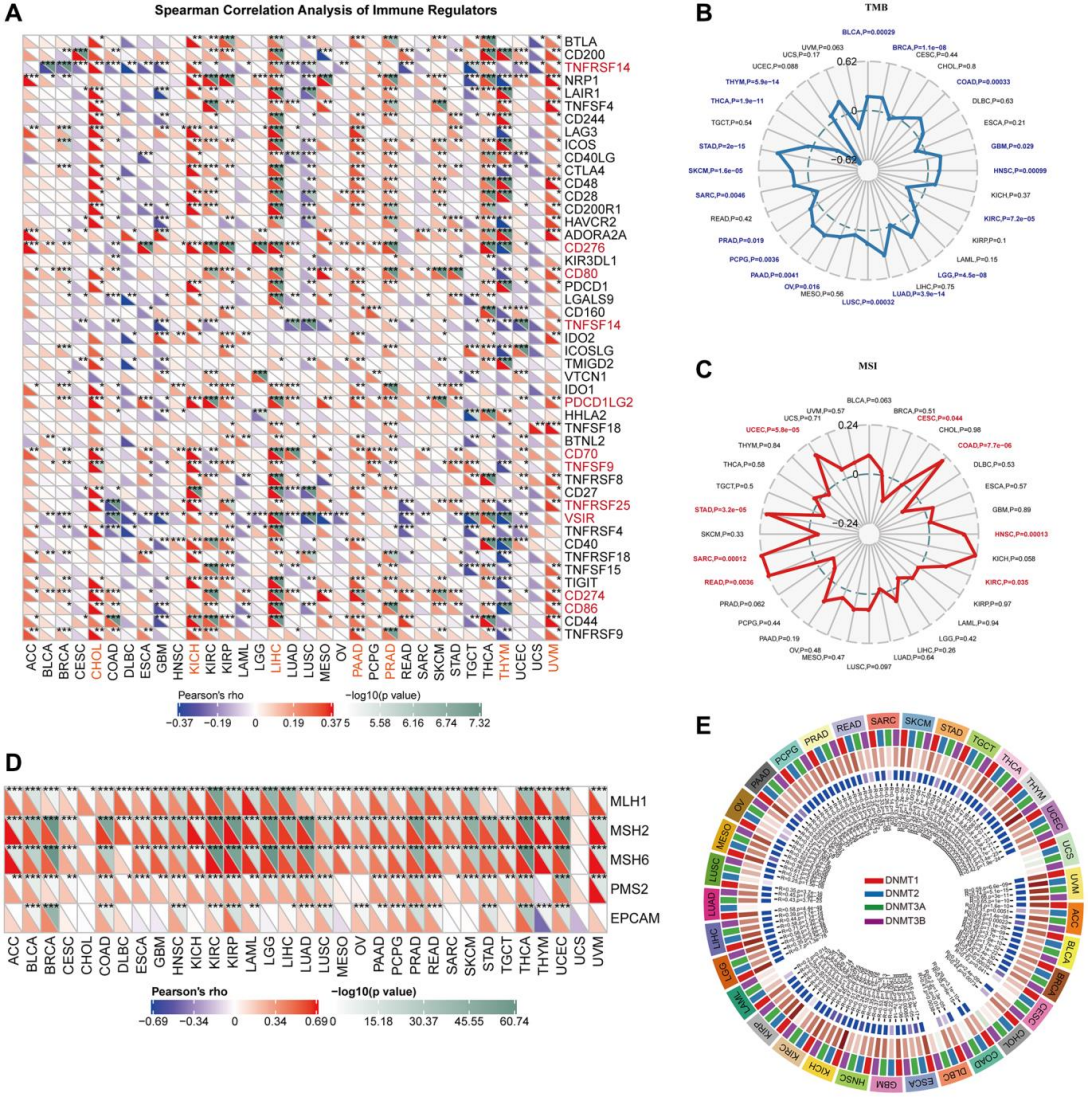
**Correlations of VRK1 expression with TME biomarkers.** (**A**) The heatmap depicts the relationships between VRK1 expression and the 47 different types of immune regulators in various cancers by Spearman correlation analysis. (**B**) Correlations between VRK1 expression and tumor mutation burden (TMB) across cancers. (**C**) Correlations between VRK1 expression and microsatellite instability (MSI) across cancers. (**D**) Correlation between VRK1 expression level and the expression of five MMR genes. The left bottom triangle in each unit represents Pearson’s correlation test coefficient of association. (**E**) Correlation between VRK1 expression level and four methyltransferase genes. (^*^P < 0.05, ^**^P < 0.01, and ^***^P < 0.001).

Figure 4A illustrates the associations between VRK1 and 47 immune regulators across different cancer types. This comprehensive analysis provides valuable insights into the potential interplay between VRK1 and immune-related genes, shedding light on its role in modulating the tumor immune microenvironment. The results found that VRK1 was related to most immune regulators in most TCGA cancers, including TNFRSF14, CD276, CD80, TNFSF14, PDCD1LG2, CD70, TNFSF9, TNFRSF25, VSIR, CD274, and CD86. Importantly, VRK1 had a robust positive relationship with most immune regulators in CHOL, KICH, LIHC, PAAD, PRAD, and UVM and a strong negative relationship with most immune regulators in THYM (Figure 4A). Additionally, our findings revealed positive correlations between VRK1 expression and tumor mutation burden (TMB) in various cancer types, including BLCA, BRCA, COAD, GBM, HNSC, LGG, LUAD, LUSC, OV, PAAD, PCPG, PRAD, SARC, SKCM, and STAD. Negative correlations were discovered in KIRC, THCA, and THYM (Figure 4B). And VRK1 expression was positively associated with MSI in COAD, HNSC, KIRC, READ, SARC, UCEC, and STAD. Negative correlations were discovered in CESC (Figure 4C). Our findings indicate that VRK1 expression might serve as a potential predictor of the responsiveness to immune checkpoint inhibitors (ICIs) in the corresponding malignancies. Furthermore, we also looked at the relationship between the expression of VRK1 and the carcinogenesis process, specifically regarding MMR deficiencies and DNA methylation of vital tumor-related genes. According to the findings, the five MMR genes were associated with VRK1 in most cancer types except for CHOL and UCS (Figure 4D). Interestingly, most cancer types had a positive connection with these MMR genes, implying that MMR regulation may play a role in carcinogenesis. Gene expression is thus regulated by DNA stability and its interactions with specific proteins. As a result, the association between the expression level of VRK1 with four DNMTs was explored. We found that VRK1 expression was remarkably correlated with these four DNMTs in almost all cancers except in UCS (Figure 4E).

### TIMER immune cell infiltration analysis

**Figure 5:**
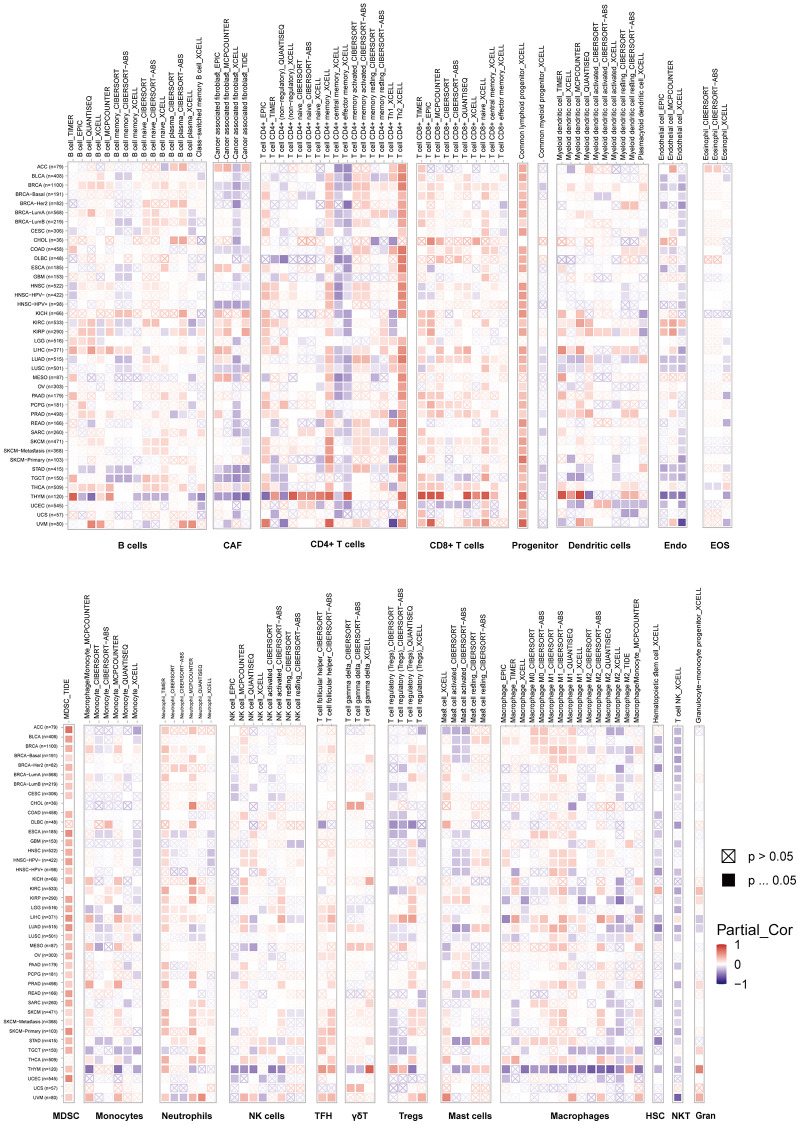
**TIMER immune cell infiltration analyses.** VRK1 expression correlates with the degree of infiltration of CD8+ T cells, CD4+ T cells, regulatory T cells (Tregs), B cells, monocytes, macrophages, NK cells, dendritic cells, mast cells, CAFs, progenitors, Endo, Eos, HSCs, TFH cells, dT cells, NKT cells, MDSCs, and neutrophils in malignancies. A positive correlation is shown in red, while a negative correlation is in blue.

To investigate the connections between VRK1 and cancer immunity, we next analyzed the relationships between VRK1 expression and immune cell infiltration, including CD8+ T cells, CD4+ T cells, Tregs, B cells, monocytes, macrophages, NK cells, dendritic cells, mast cells, CAFs, progenitors, Endo cells, HSC, Tfh cells, γ/δ T cells, NKT cells, MDSCs, neutrophils (Figure 5). We found that VRK1 was positively associated with the common lymphoid progenitor and MDSC in most TCGA cancers. In contrast, VRK1 expression was negatively related to the HSC and NKT in most TCGA cancers. Simultaneously, VRK1 showed a negative association with CAF, endo, HSC, and NK cells in some specific tumors. In general, VRK1 was correlated with the level of immune infiltration of many kinds of infiltrating cells. Moreover, by integrating ImmuneScore, EstimateScore, StromalScore, and neoantigen data, we observed that VRK1 was associated with immune infiltration in certain cancer types. This suggests that VRK1 may play a role in modulating the tumor microenvironment and influencing the immune response in these specific cancers. Thus, the findings indicated that VRK1 might interact with immune cells to influence cancer emergence, prognosis, and treatment.

### Predictive analysis of VRK1 in pan-cancer

**Figure 6:**
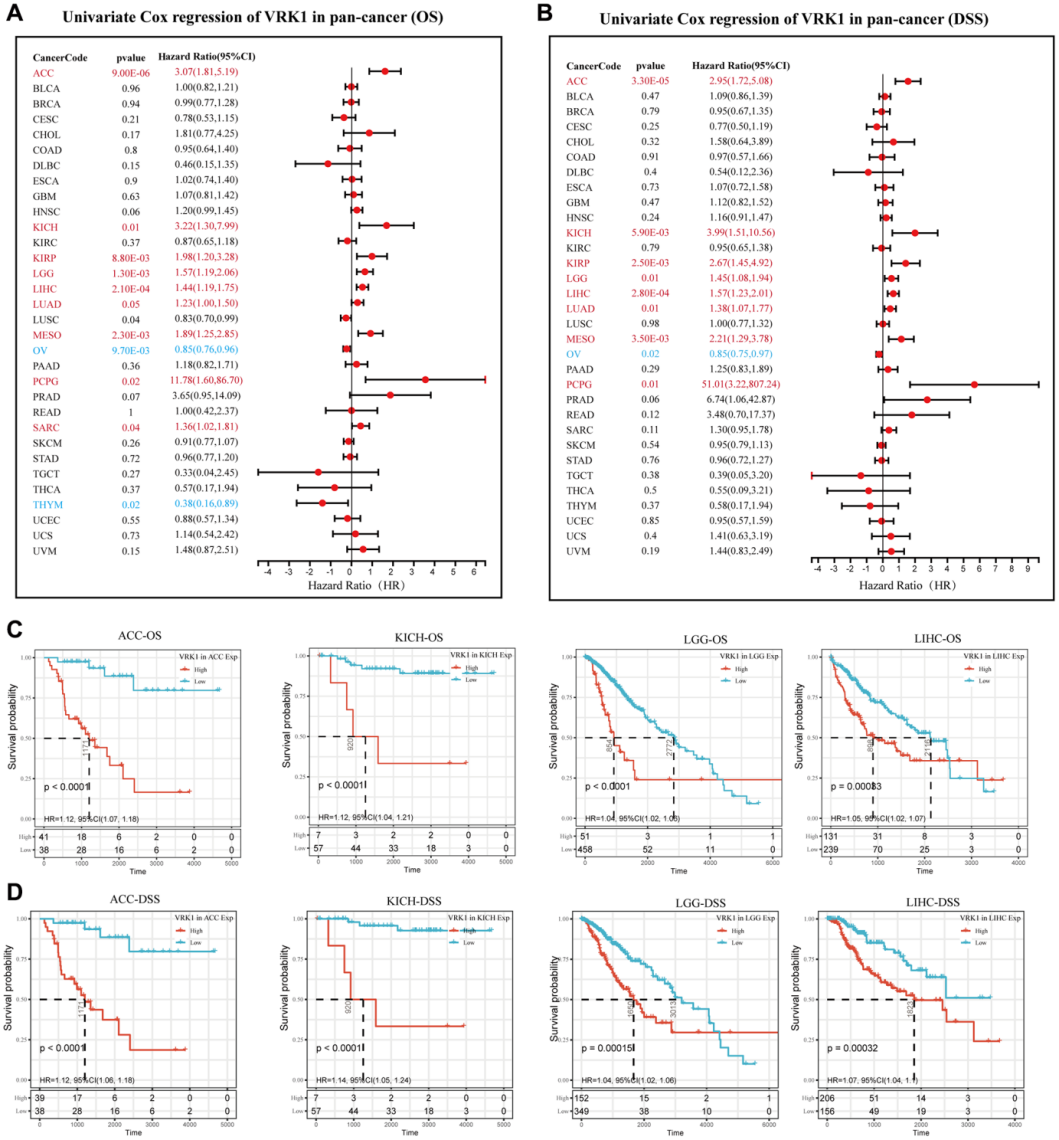
**Predictive analysis of VRK1 in pan-cancer.** (**A**) The forest plot shows the association between VRK1 expression and cancer OS by the univariate Cox regression method. (**B**) The forest plot shows the association between VRK1 expression and cancer DSS by the univariate Cox regression method. (**C**) Kaplan-Meier OS curves of VRK1 in ACC, KICH, LGG, and LIHC. (**D**) Kaplan-Meier DSS curves of VRK1 in ACC, KICH, LGG, and LIHC.

To gain further insights into the predictive potential of VRK1 in various cancers, we analyzed the prognostic indicators of 33 different cancer types using the Kaplan-Meier method and univariate Cox regression analysis. The forest plot results (Figure 6A) showed that the downregulation of VRK1 expression had relationships with OS time prolongation in ACC, KICH, KIRP, LGG, LIHC, LUAD, MESO, PCPG, and SARC. The upregulation of VRK1 expression was related to the time delay of OS in OV and THYM. In addition, we found that the downregulation of VRK1 expression had unique relationships with DSS time prolongation in ACC, KICH, KIRP, LGG, LIHC, LUAD, MESO, and PCPG. The upregulation of VRK1 expression was only related to the time delay of DSS in OV (Figure 6B). Additionally, we analyzed the Kaplan-Meier survival curves of ACC, KICH, LGG, and LIHC. The results indicated that higher VRK1 expression was significantly associated with poorer survival outcomes in these cancers, namely ACC, KICH, LGG, and LIHC (Figure 6C, 6D). These findings underscore the potential prognostic relevance of VRK1 in these specific cancer types and emphasize its role as a predictive marker for patient survival.

### Correlation analysis with methylation profile

**Figure 7:**
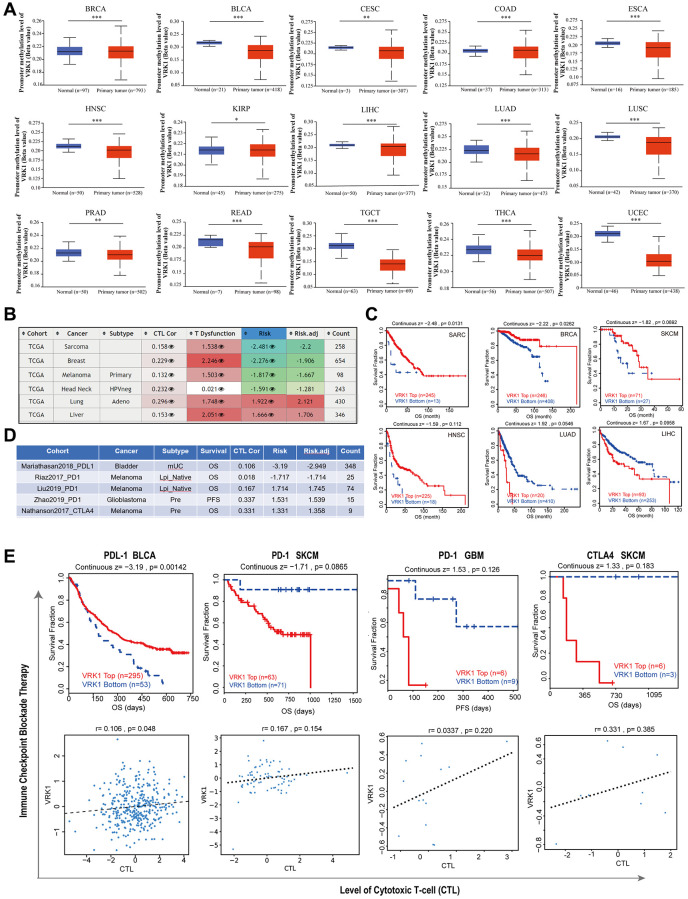
**Correlation analysis with methylation profile.** (**A**) Boxplots showing differential VRK1 methylation levels (beta values) between tumors and adjacent tissues across the TCGA dataset. (**B**) Heatmap showing the roles of VRK1 methylation in cytotoxic T-cell levels (CTLs), dysfunctional T-cell phenotypes, and risk factors of TCGA cancer cohorts. (**C**) Kaplan-Meier curves of OS differences between TCGA cancer cohorts with high methylation levels and those with low methylation levels of VRK1. (**D**, **E**) The upper panel displays Kaplan-Meier curves illustrating the survival ratios of cancer cohorts with high and low expression levels of VRK1 as a measure of the immunotherapeutic response (immune checkpoint blockade). The lower panel presents the correlation between VRK1 expression levels and cytotoxic T-cell levels (CTL) in these cohorts. Only statistically significant differences between the cohorts in TCGA cancers are included in the analysis.

Promoter methylation analysis revealed that VRK1 exhibited hypermethylation in BRCA, COAD, and KIRP, suggesting potential epigenetic regulation of VRK1 in these particular cancer types. At the same time, it was hypomethylated in various cancer types, including BLCA, CESC, ESCA, HNSC, LIHC, LUAD, LUSC, PRAD, READ, TGCT, THCA, and UCEC (Figure 7A). Our analysis revealed a positive correlation between the hypomethylation of VRK1 and shorter survival durations in SARC, BRCA, SKCM, and HNSC. Conversely, hypomethylation of VRK1 was associated with a favorable prognosis in LUAD and LIHC (Figure 7B, 7C). Furthermore, we observed that decreased expression levels of VRK1 were linked to improved clinical outcomes of ICB therapy (PD-1, PD-L1, or CTLA4) in SKCM and GBM. Patients with lower VRK1 expression levels had more extended survival periods than those with high VRK1 expression levels. On the other hand, high VRK1 expression was linked to clinical benefits in BLCA patients receiving PDL-1 ICB therapy, resulting in increased survival durations compared to BLCA cohorts with lower VRK1 expression levels. Furthermore, increased expression levels of VRK1 in SKCM, GBM, and BLCA were positively associated with the level of CTL (Figure 7D, 7E).

### Gene set enrichment analysis of VRK1 in pan-cancer

**Figure 8:**
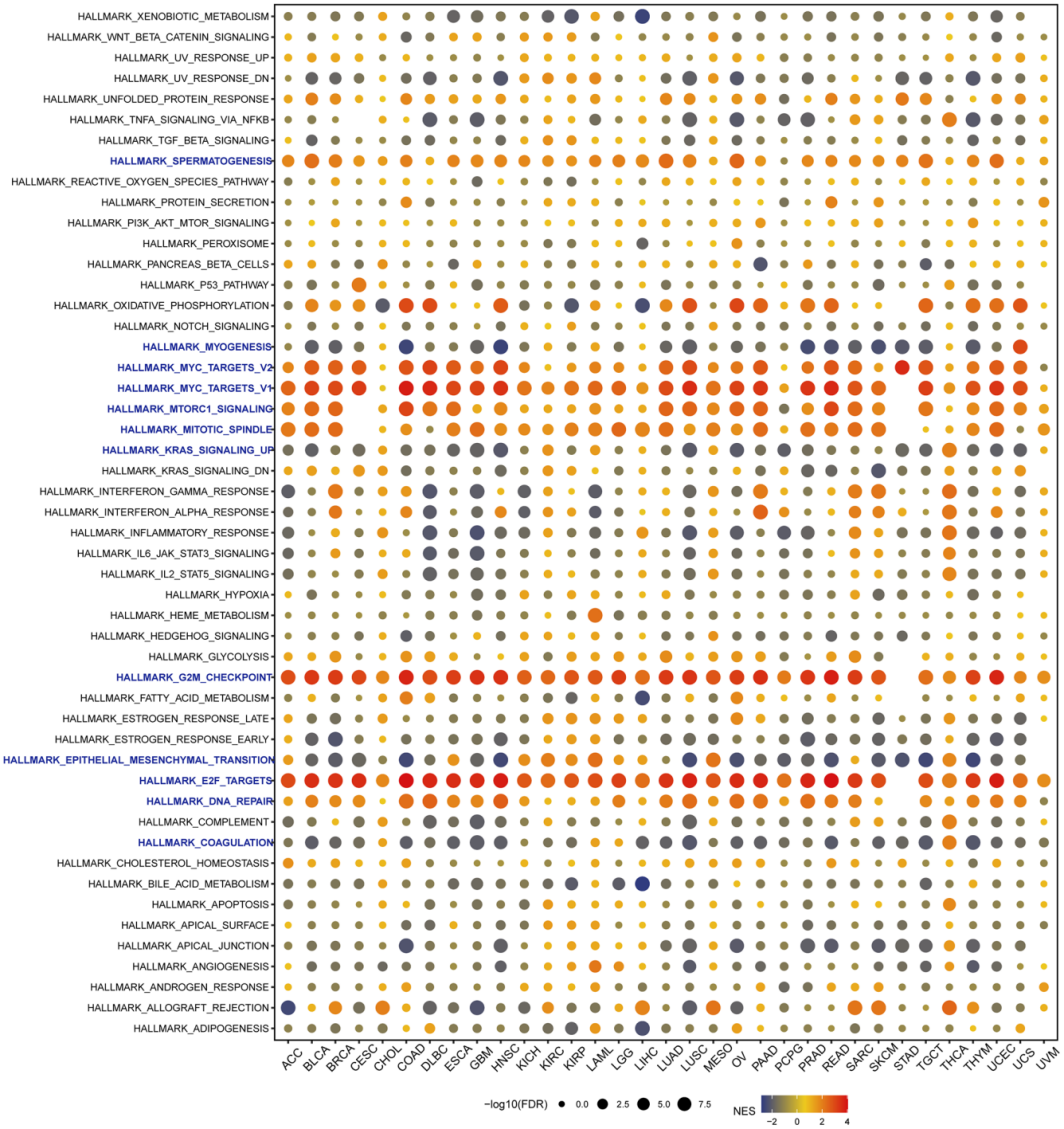
**Gene set enrichment analysis (GSEA) of VRK1 in pan-cancer.** The circle size represents the FDR value of the enriched term in each cancer, and the color indicates the normalized enrichment score (NES) of each term.

Given the substantial prognostic significance of VRK1 in various cancers, we investigated to explore the underlying biological processes or pathways associated with VRK1, aiming to elucidate its potential mechanisms of action. As part of this study, we analyzed a hallmark gene set comprising marker genes that define tumor physical status and progression. To explore the functional implications of VRK1 expression, we identified differentially expressed genes (DEGs) between low- and high-VRK1 subgroups in each cancer. We displayed the enrichment status of VRK1 in each pathway (Figure 8). We found VRK1 expression was associated with immune-related pathways, such as myogenesis, myc-targets-V1/V2, KRAS-signaling-up, G2-checkpoint, epithelial mesenchymal-transition, E2-targets, and coagulation in most cancers, which uncovered a potential association between VRK1 expression and immune activation in the tumor microenvironment (TME). In addition, G2-checkpoint and E2-targets were significantly enriched in high-VRK1 patients except for STAD. Studies have reported that EMT plays an essential role in the molecular mechanism and treatment of tumor progression and metastasis [26], which implies that VRK1 may play a critical role in cancer oncogenesis and development by participating in EMT.

### Suppression of VRK1 inhibited invasion and migration of HCC cell lines

**Figure 9:**
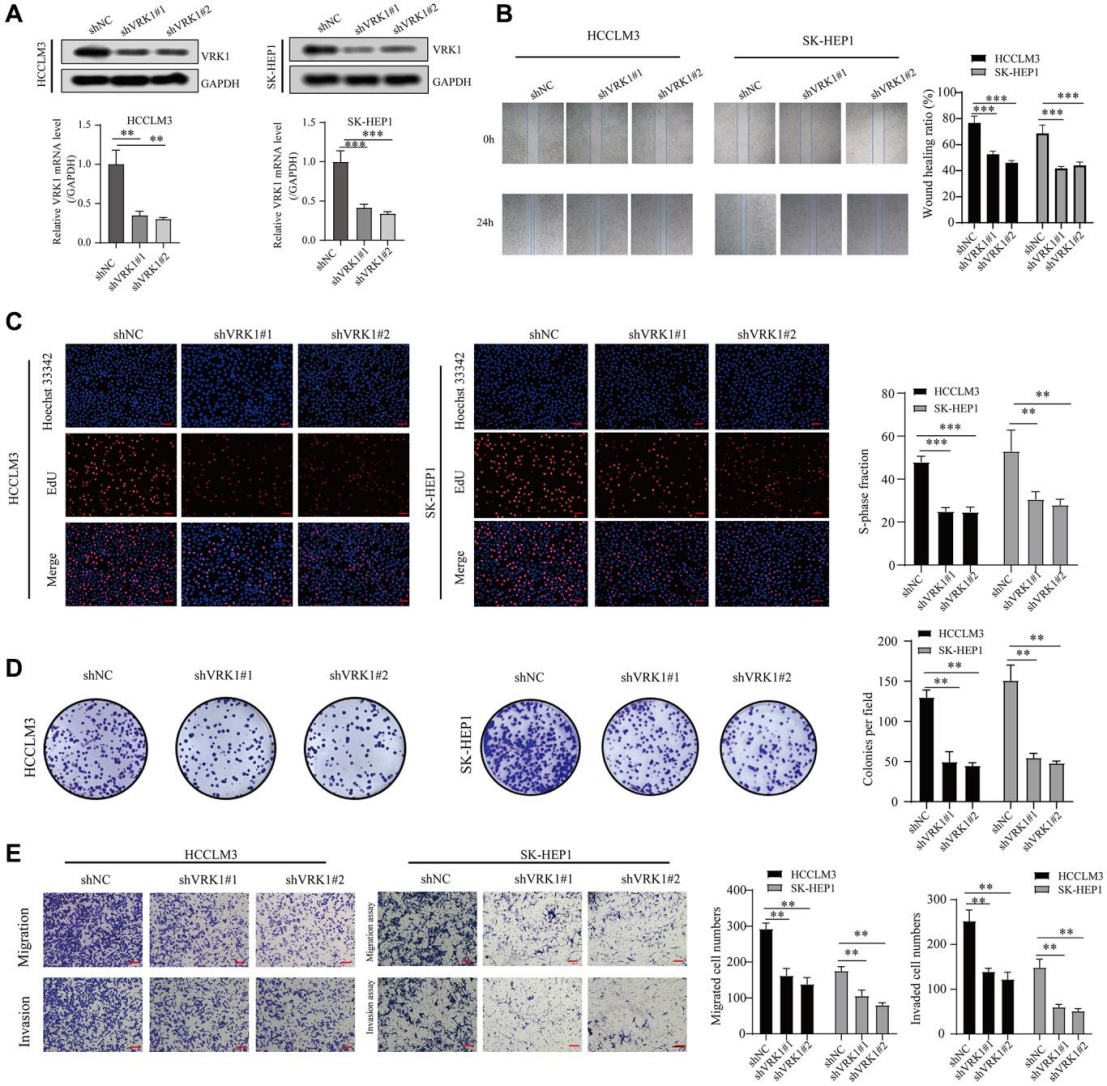
**VRK1 promotes HCC cell proliferation and invasion.** (**A**) The protein and mRNA expression levels of VRK1 in HCC cells after transfection with shVRK1 or shNC. (**B**) Statistical plots and representative images of the wound healing assay in HCC cells transfected with shVRK1. (**C**) Representative images and quantification of Edu assay in HCC cells transfected with shVRK1. (**D**) Colony formation assays detecting the cell proliferation in HCC cells transfected with shVRK1. (**E**) Transwell assays show the suppressed migration and invasion ability of HCC cells transfected with shVRK1 (^*^P < 0.05, ^**^P < 0.01, and ^***^P < 0.001).

We explored the effect of VRK1 on cell proliferation, invasion, and migration through a series of cell function experiments. First, we verified the efficiency level of knocking down VRK1 by qRT-PCR and western blotting experiments. The results of our study found downregulating VRK1 significantly reduced both mRNA and protein expression levels of VRK1 HCCLM3 and SK-HEP1 cells (Figure 9A). Next, EdU and colony formation experiments indicated that shVRK1 significantly decreased the proliferative ability of HCCLM3 and SK-HEP1 cells (Figure 9C, 9D). In addition, wound healing and transwell assays also indicated that the knockdown of VRK1 significantly weakened the migrative and invasive abilities of HCCLM3 and SK-HEP1 cells (Figure 9B, 9E). Thus, we believed that silencing VRK1 inhibited HCC cells proliferation and invasion.

## DISCUSSION

It is known that VRK1 plays an integral role in the regulation of the cell cycle. High expression of VRK1 appears in different tumors and promotes tumor progression [27, 28]. However, to date, no study has analyzed the role of VRK1 in pan-cancer analysis. By analyzing these large-scale datasets, we aimed to gain a comprehensive understanding of VRK1’s expression patterns in different cancers, shedding light on its potential roles and implications in cancer development and progression. Our findings revealed that VRK1 expression was markedly upregulated in a majority of cancers. And notably, VRK1 was only lowly expressed in LAML and TGCT. To validate the outcomes of the bioinformatic analyses, we conducted a clinical sample test. Our findings confirmed that VRK1 expression was indeed elevated in LIHC tissues at both the mRNA and protein levels when compared to corresponding adjacent normal liver tissue, aligning with the results obtained from the bioinformatic analyses. This convergence of findings further strengthens the evidence supporting the dysregulation of VRK1 in LIHC and reinforces its potential role as a relevant biomarker in liver cancer. These results suggest that VRK1 is overexpressed in various cancers and may be involved in tumor initiation and progression. The study identified VRK1 as a novel neuroblastoma tumor progression marker that regulates cell proliferation [29]. As an early response gene, its loss causes a block in cell cycle progression [16], suggesting that VRK1 plays an important part.

Genetic mutations drive normal cells through the stages of hyperplasia and dysplasia, ultimately leading to invasive cancer and potential metastasis [30]. Gene mutations are common in tumors and play a vital role in the development of tumors [31]. And the build-up of genetic changes propels the advancement of normal cells from hyperplasia and dysplasia stages toward invasive cancer and, eventually, metastatic malignancy [30]. Therefore, analyzing gene alterations of known oncogenes could offer additional insights into the functions of these genes in the progression of cancer. We examined the frequency and types of genetic alterations of VRK1 in various cancer types. We observed that deep alteration was the most common genetic alteration of VRK1, whereas structural variant was less frequent. Furthermore, we found that VRK1 mutations do not occur in every tumor, such as LAML, THCA, UCS, and UVM. The cancer with the highest mutation frequency of VRK1 was UCEC, which exceeded 4% and mainly was the “Mutation” type. These research findings contribute to a deeper understanding of VRK1, in the development of cancer. Furthermore, they provide valuable insights for the formulation of gene therapy and personalized treatment strategies.

More importantly, complicated patterns of genetic mutations are frequently associated with genomic instability in the later stages of the disease, which may obscure the events underlying the malignant transformation. To thoroughly understand how malignant tissues evolve, it is vital to evaluate all stages of disease progression. Indeed, the intriguing findings that genetic alterations of VRK1 are associated with better prognoses in terms of OS, DSS, and PFS raise compelling possibilities. The comprehensive expression profiles of VRK1 across primary tumors, pathological stages, and metastatic conditions further suggest its involvement at various stages of tumor progression. Consequently, VRK1 emerges as an appealing candidate for potential cancer therapies. Nonetheless, it is crucial to acknowledge that cancer development and progression are intricate processes governed by the interplay of multiple genes. Frequently, co-occurrences of gene alterations are observed, and these co-drivers act in tandem with primary genetic drivers to promote tumor progression and potentially impact therapeutic responses. Thus, while VRK1 holds promise as a target for cancer therapy, considering its interactions with other genes and pathways will be essential to develop effective and comprehensive treatment strategies. A holistic approach considering the complex genetic landscape is imperative to unlock the full potential of VRK1 as a therapeutic target in cancer [32, 33]. Hence, by analyzing the co-occurrence of gene expression and alterations, we have identified potential functional partners of VRK1 in cancer. Interestingly, we have observed a strong correlation between VRK1 and genes such as IGHJ2, INAFM1, KBTBD13, DACT3-AS1, LOC93429, RNU6-66P, EML2-AS1, and MEIOSIN across different cancer types. Moreover, the high frequency and consistent pattern of co-occurring genetic alterations in these genes strongly suggests their involvement as functional partners in the oncogenic role of VRK1 in various cancers.

Additionally, the correlation analysis conducted between VRK1 and the pan-cancer immunomodulatory factors indicates a strong correlation between VRK1 expression and the expression of immunomodulatory genes, particularly in LIHC and THYM. The results suggest a potential role for VRK1 in regulating the immune response in these cancer types. And in LIHC, VRK1 has positively correlated with almost all immune checkpoints except for IDO2 and ICOSLG. The findings suggest that VRK1 may have played a crucial role in advancing and predicting cancer through its interaction with the microenvironments associated with the disease. In addition, TMB, as a predictive biomarker in solid tumors has the potential to serve as a predictive biomarker for immunotherapy outcomes in solid tumors, enhancing the accuracy of predictions and potentially broadening the pool of eligible patients for immune checkpoint inhibitor treatment [34]. Microsatellite instability (MSI) is characterized by frequent variations in short repetitive DNA sequences and single nucleotide substitutions due to a deficiency in DNA mismatch repair (MMR), resulting in a hypermutator phenotype. In addition, MSI status is a known predictor of cancer’s response or resistance to certain chemotherapies [35]. Subsequently, the correlation between VRK1 and TMB was observed in 18 types of cancer. In 8 types of cancer, it was observed between VRK1 and MSI. These findings imply that VRK1 has the potential to serve as a novel biomarker for predicting the response to ICI therapy in specific cancer types. Our study revealed a significant correlation between immune infiltration in cancers and the expression of VRK1. In most tumors, VRK1 was positively associated with the infiltration of progenitors of lymphoid, and MDSC, suggesting that VRK1 was likely to affect tumor development and prognosis by impacting the tumor microenvironment (Figure 5). Therefore, we speculate that the potential association between VRK1 and these immune regulators is promising for further investigation.

In this study, VRK1 was identified as a risk factor in 9 cancer types and a protective factor in 2 cancers. These results highlight the significant role of VRK1 in predicting the prognosis of cancer patients, making it a promising biomarker for assessing patient outcomes. These findings align with conclusions from previous research, further supporting the potential clinical relevance of VRK1 as a prognostic indicator in various cancer types [6, 36–38], making our results more reliable. Together, VRK1 could serve as a promising prognostic marker for cancer patients. Our study analyzed the expression patterns and predictive values of VRK1 in pan-cancer using multiple databases. We observed that VRK1 expression was increased in most cancer types and that high levels of VRK1 were associated with poor survival outcomes and tumor progression in some cancers. Furthermore, we discovered that VRK1 was closely linked to TME, tumor infiltration immune cells, immune subtypes, and biomarkers of ICIs, providing new insights into the role of VRK1 in tumor immunity. These findings may be beneficial in identifying novel therapeutic targets and predictive biomarkers for immunotherapy. Notably, this study is to validate the differential expression of VRK1 in HCC tissues and investigate the role of VRK1 in HCC cell proliferation and invasion. Our findings provide a promising foundation for developing biomarker-targeting therapies in HCC. Admittedly, our research has several limitations. Although we predict the correlation between VRK1 and some signal pathways through functional enrichment analysis, we have not conducted experimental verification on these signal pathways in this study, and the specific molecular mechanism is unclear. *In vivo* animal experiments will remain essential for future research endeavors. In addition, our research collected sequencing data from the open database for analysis. Although the data is standardized, there are still inevitable systematic deviations. Nevertheless, this pan-cancer study provides a deeper understanding of the role of VRK1 in the functional nucleus of different tumors.

## CONCLUSION

In summary, our comprehensive pan-cancer analysis of VRK1 has revealed its potential as a valuable cancer prognosis biomarker. Moreover, we identified a close association between VRK1 expression and the tumor microenvironment (TME), tumor-infiltrating immune cells, immune subtypes, and immune checkpoint inhibitors (ICIs) biomarkers. These findings offer new insights into VRK1’s role in tumor immunity, presenting opportunities for identifying novel therapeutic targets and predictive biomarkers for immunotherapy. Furthermore, our study successfully validated the differential expression of VRK1 in LIHC tissues and investigated its impact on the proliferation and invasion of HCC cells. This provided the initial groundwork for the development of biomarker-targeting therapies in HCC. Altogether, our research sheds light on the multifaceted role of VRK1 in cancer and presents a promising avenue for future investigations and therapeutic advancements in the field of cancer treatment.

## Supplementary Materials

Supplementary Figure 1

Supplementary Table 1
