# Supplementary material for: Comprehensively prognostic and immunological analysis of VRK Serine/Threonine Kinase 1 in pan-cancer and identification in hepatocellular carcinoma
Source: Aging (Albany NY). 2023 Dec 28;15(24):15504–24. doi: 10.18632/aging.205389 (PMC10781469; doi:10.18632/aging.205389)
Supplement: Supplementary Figure 1 [file aging-15-205389-s001.pdf]

SUPPLEMENTARY FIGURE

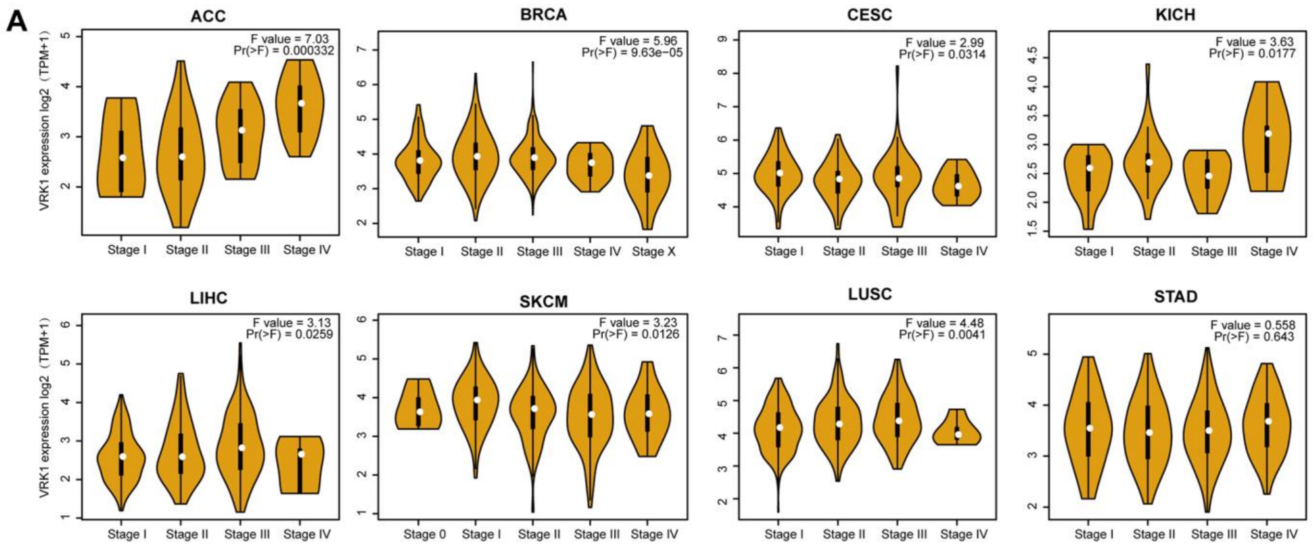

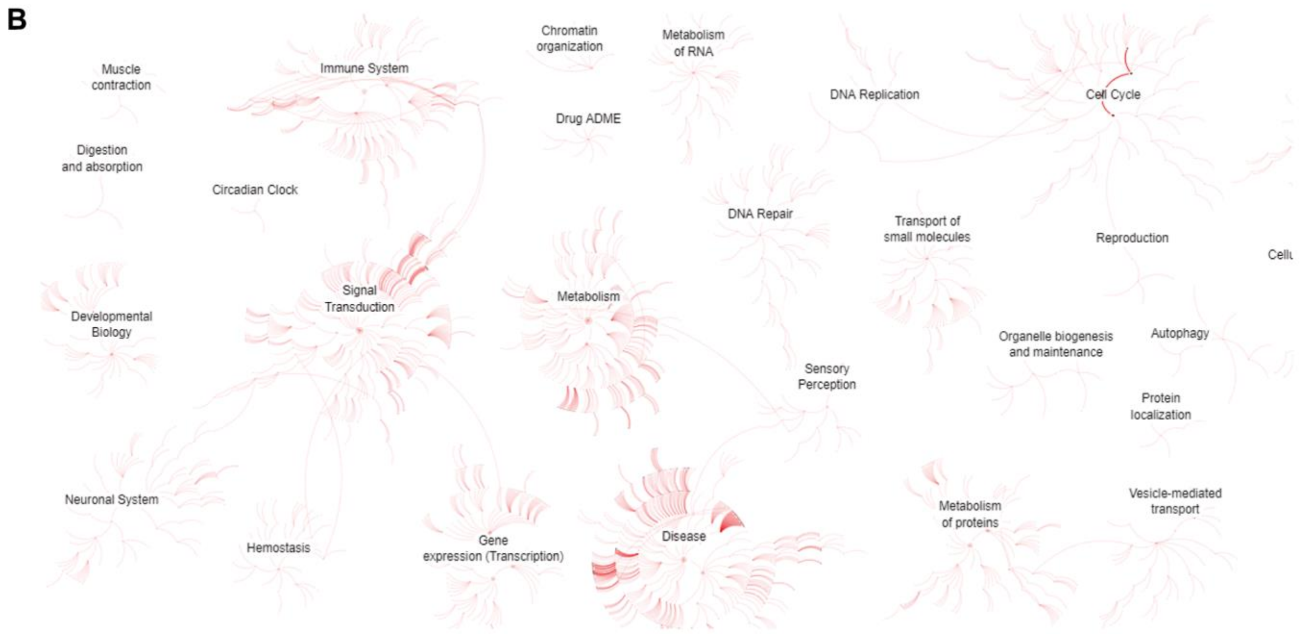

Supplementary Figure 1. (**A**) Violin plots showing differential VRK1 expression levels between pathological stages in various cancers. (**B**) Pathway analysis for genes co-expressed with VRK1 using Reactome. Pink depicts pathways containing genes co-expressed with VRK1.
